# The utilization of medical information systems in future radiation disasters: reflections on a comparison of experiences of utilization between Japan and Taiwan

**DOI:** 10.3389/fpubh.2023.1288315

**Published:** 2023-12-14

**Authors:** Chie Kushima, Naoya In, Fu-Chih Lai, Hiroyuki Hanada, Toshiko Tomisawa

**Affiliations:** ^1^Department of Nursing Sciences, Hirosaki University Graduate School of Health Sciences, Hirosaki, Japan; ^2^Taipei Medical University College of Nursing, Taipei, Taiwan; ^3^Hirosaki University Graduate School of Medicine, Hirosaki, Japan

**Keywords:** medical information systems, radiation disaster, complex disaster, Japan, Taiwan

## Introduction

The UN Economic and Social Commission for Asia and the Pacific (ESCAP) has highlighted the use of information and communications technology during all phases of disaster risk management as providing “substantial opportunities to reduce disaster risks, enhance coping capabilities, and provide inclusive preparedness and response” (1). In March 2011, Japan experienced the Great East Japan Earthquake, which underlined major problems with information and communication technology, especially in the Fukushima Daiichi nuclear power plant accident. Narcyz et al. (2) reported the current landscape of research dealing with the role of e-government in disaster management, but no study has mentioned radiation disasters.

In Taiwan, the hierarchy of the medical care delivery system is based on the respective medical functions and services provided (3). Taiwan has achieved remarkable management of several disasters through its e-governance, which is based on the NHI medical information system. These achievements have illuminated the implications of the utilization and integration of medical information systems for disaster management. In March 2023, an exploratory visit to Taiwan was embarked upon to obtain greater insight into the actual practice of utilizing the medical information system for future radiation disaster preparedness, which can serve as the underpinning of integration, coordination, and improvement for the use of the Japanese medical information system for disaster management.

## Differences and similarities between Japan and Taiwan in the disaster utilization of medical information systems

The Japanese universal health insurance program was introduced in 1961 (4). However, medical information is managed by hospitals or clinics and is not centrally controlled. Under such circumstances, inquiries into medical information rely on the input of hospitals or clinics, which has hindered the accessibility, availability, and efficiency of access to information needed by the Japanese central government. This problem has influenced e-governance during disaster management.

During disasters or health emergencies, the system is divided into separate sub-systems. For example, the government uses the Emergency Medical Information System (EMIS) during disasters and the Gathering Medical Information System (G-MIS) for COVID-19 during health emergencies relating to the COVID-19 pandemic (5).

EMIS is a system in which local governments, hospitals, and healthcare centers in the affected areas report on the status of the disaster, victims, and medical resources. The local governments and hospitals outside the affected areas can access the system to acquire information on the situation. The Disaster Medical Assistance Team (DMAT) accesses EMIS to collect information and shares this information with the Medical Air Transport Tracking System (MATTS) for transporting victims. When the DMAT arrived at the Fukushima Daiichi nuclear power plant during the 2011 Great East Japan Earthquake, they reported that the entire disaster response headquarters at the Fukushima Prefectural Government was in a chaotic state, with uncontrolled and disorganized information (6, 7). Surveys in the Fukushima and Miyagi areas, which were damaged by the tsunami, indicated that even the government was unable to ascertain the numbers and gathering locations of disaster victims due to the large size of the affected areas and cut-off communications. This led to many isolated areas where it took longer for victims to access available support, including medication shortages in hospitals and pharmaceutical companies (8).

In Taiwan, the universal healthcare system was introduced in 1995, funded by the NHI system for the entire population (9), and an NHI card was issued to all insured individuals. Through this system, the Ministry of Health and Welfare (MOHW) is able to manage the medical information of the Taiwanese population, which includes demographic information, medical history, hospital visits, medication prescription history, vaccination status, and healthcare resources used. Using the aforementioned infrastructure and mechanisms during the COVID-19 pandemic, a comprehensive picture of the pandemic status of the population and of the availability and accessibility of hospital beds, medical equipment, and resources could be sketched instantly based on the information obtained from the NHI medical information system. That information can be communicated and coordinated with the multidisciplinary team for all phases of disaster management.

The utilization of this NHI medical information system was observed during our visit to Linkou Chang-Gung Medical Hospital (CGMH) and Kaohsiung Medical University Chung-Ho Memorial Hospital (KMUCHMH). Those observations can be taken as applied examples of how the medical information systems were used and integrated vertically from hospitals to the Regional Emergency Management Operations Centers (REMOCs) and NHI with information on bed, equipment, and manpower availability; and horizontally from hospitals to hospitals and local government with information on client situation, distribution, and healthcare needs. This information was integrated and interactively communicated in the dual directions of bottom-up and top-down for sharing and communication of information during disaster management.

The Taiwan MOHW, the equivalent of Japan's Ministry of Health, Labor, and Welfare (MHLW), is responsible for public health, social welfare, and assistance (10), and consolidates information on serious accidents and disasters in Taiwan. A critical role and function of the MOHW in disaster management is surveillance of the national healthcare system, which involves collecting and analyzing all medical information for decision-making support and improving the efficiency of the emergency medical response. It can conduct real-time video conferences with local governments, REMOCs, other government agencies, hospitals and clinics to communicate, integrate and coordinate medical information.

The Taiwan MOHW collects medical information, monitors medical resource capabilities, and instantly updates that information daily. During disasters, the MOHW immediately convenes personnel, issues instructions for emergency response, and coordinates and supports medical resources. The Taiwanese medical information system facilitates the MOHW in coordinating and collecting medical information, supports decision-making in the distribution and coordination of resources, makes decisions in the coordination of emergency medical rescue resources across regions, coordinates means of patient transport and accommodation for disaster victims, and improves the efficiency of the emergency medical response under the multidisciplinary approach to disaster management. This infrastructure and associated mechanisms have shown their applicability during a major gas explosion in Kaohsiung in 2014 and the Formosa water park powder explosion in 2015. Based on this infrastructure, the central government was also able to access the Kaohsiung EMOC in real time, which allowed them to monitor medical information and resources, reducing the time taken to transport patients and provide medical treatment.

The Situation Center at the MOHW has an information platform where a variety of information is aggregated and displayed. For example, emergency response hospital record data; data from the SW (volunteer services and shelter information), MA (Emergency Medical, Medical Affairs, and AED information), NHII (health insurance claims data), CDC (infectious disease information), and FDA (disaster medical supplies); daily blood reserve data; and antidote data in Taiwan are all coordinated, managed, and monitored by the MOHW. The information aggregated in this system is analyzed and shared with central, municipal, and local governments to direct the response and act upon the disaster from multidimensional perspectives. The results of a comparative analysis of differences and similarities in the utilization of medical information systems in disasters between Japan and Taiwan, as mentioned above, are elaborated in Table 1.

**Table 1:** Utilization differences and similarities of medical information system in disaster between Japan and Taiwan.

			**JAPAN (ex.) Fukushima Daiichi nuclear plant accident**	**TAIWAN (ex.) Kaohsiung gas explosion**
Differences	Medical information system during disasters or accidents	Patient	1. Local governments, hospitals and health care center report to local DMAT through EMIS. 2. Local DMAT collaborates with Central DMAT. 3. Central government can access information Central DMAT has.	Centralized management by the NHI system (Central and local governments and hospitals can access)
		Medical response capacity and critical resources		Centralized management by the Situation Center (Central and local governments and hospitals can access)
	Medical information system on a daily basis	Patient	Collected and managed by each hospital (Central and local governments do not manage or integrate)	The same as during disasters or accidents
		Medical response capacity and critical resources	Collected and managed by each hospital (Central and local governments do not manage or integrate)	
Similarities	Medical insurance		Medical insurance for the whole nation	Medical insurance for the whole nation

## Radiation disaster preparedness in Taiwan

Taiwan currently has one nuclear power plant in operation, and the other two are decommissioned. In radiation disaster operations, levels of radiation disaster are categorized (from the least hazardous to the most hazardous) as 1, 2, and 3. Medical information collected from responding hospitals is not only shared with the local EMOC but also with the central government agencies of the MOHW, Atomic Energy Council, and Nuclear Safety Commission for radiation disaster management.

KMUCHMH in Southern Taiwan is a designated hospital responsible for radiation disasters with a Radiation Emergency Medical Center (REMC) system. The hospital visualizes red-line routes for patients requiring isolation to pass through to avoid contact contamination with other patients and medical personnel along with monitoring of radiation doses. Additionally, the “Orange Code” is triggered to alert staff from various departments with multiple specialties in response to radiation disasters immediately. The aforementioned activation system, response infrastructure, and mechanisms are used in constant training, with simulations and exercises/drills conducted regularly. Linkou CGMH is the largest hospital in Northern Taiwan and, like KMUCHMH, has been designated as the hospital responsible for radiation disasters. With 9,000 medical personnel, it has the capacity to treat approximately 200,000 emergency patients annually and is expected to receive many cases of exposure to radiation disasters.

Although Taiwan is at risk of radiation disasters without having experienced any, those two hospitals conduct regular radiation disaster drills while recognizing the urgent need for education and training in radiation disasters, having learned from the Japanese experience. The integrated infrastructure and mechanisms of medical information systems are also embraced in radiation disaster drills in order to respond to disasters effectively.

## Discussion

In Japan, cloud systems for disaster management have been developed but operate separately at the central and local levels (11, 12). Reflections from the visit to Taiwan highlight the importance of integrating the central, municipal, and local levels of medical information systems to provide guidelines in centralized disaster management. When a rapid response is required for a radiation disaster, with medical information that has been centrally managed and updated daily, along with information on the affected population, medical response capacity, and critical resources available at the impact time in a disaster, the initial response can be accelerated effectively. Thus, the integration issue serves as the critical factor in resolving the difficulties of the initial response to disasters; this requires changes of policies and e-governance approaches in Japan. In addition to vertical integration between central and local levels, Japan's MHLW oversees natural disasters. In contrast, the Nuclear Regulation Authority oversees radiation disasters, which highlights the need for horizontal integration; this will enable the use of e-governance in radiation disaster management. The reflections gained and presented in this report will provide insights for leaders and administrators in promoting and fulfilling e-governance for disaster management and resilience development for future disasters.

## Author contributions

CK: Writing – original draft. NI: Writing – review & editing. F-CL: Writing – review & editing. HH: Writing – review & editing. TT: Writing – review & editing.
